# Development, refinement, and validation of an equine musculoskeletal pain scale

**DOI:** 10.3389/fpain.2023.1292299

**Published:** 2024-01-19

**Authors:** Ulrike Auer, Zsofia Kelemen, Claus Vogl, Stephanie von Ritgen, Rabea Haddad, Laura Torres Borda, Christopher Gabmaier, John Breteler, Florien Jenner

**Affiliations:** ^1^ Anaesthesiology and Perioperative Intensive Care Medicine Unit, Department of Companion Animals and Horses, University of Veterinary Medicine Vienna, Vienna, Austria; ^2^ Equine Surgery Unit, Department of Companion Animals and Horses, University Equine Hospital, University of Veterinary Medicine Vienna, Vienna, Austria; ^3^ Department of Biomedical Sciences, Institute of Animal Breeding and Genetics, University of Veterinary Medicine Vienna, Vienna, Austria

**Keywords:** chronic pain, pain scale, musculoskeletal pain, discomfort, horse, equine

## Abstract

Musculoskeletal disease is a common cause of chronic pain that is often overlooked and inadequately treated, impacting the quality of life of humans and horses alike. Lameness due to musculoskeletal pain is prevalent in horses, but the perception of pain by owners is low compared with veterinary diagnosis. Therefore, this study aims to establish and validate a pain scale for chronic equine orthopaedic pain that is user-friendly for horse owners and veterinarians to facilitate the identification and monitoring of pain in horses. The newly developed musculoskeletal pain scale (MPS) was applied to 154 horses (mean age 20 ± 6.4 years SD) housed at an equine sanctuary, of which 128 (83%) suffered from chronic orthopaedic disease. To complete the MPS, the horses were observed and videotaped from a distance while at rest in their box or enclosure. In addition, they received a complete clinical and orthopaedic exam. The need for veterinary intervention to address pain (assessed and executed by the sanctuary independent from this study) was used as a longitudinal health outcome to determine the MPS’s predictive validity. To determine the interrater agreement, the MPS was scored for a randomly selected subset of 30 horses by six additional blinded raters, three equine veterinary practitioners, and three experienced equestrians. An iterative process was used to refine the tool based on improvements in the MPS’s correlation with lameness evaluated at the walk and trot, predictive validity for longitudinal health outcomes, and interrater agreement. The intraclass correlation improved from 0.77 of the original MPS to 0.88 of the refined version (95% confidence interval: 0.8–0.94). The refined MPS correlated significantly with lameness at the walk (*r* = 0.44, *p* = 0.001) and trot (*r* = 0.5, *p* < 0.0001). The refined MPS significantly differed between horses that needed veterinary intervention (mean MPS = 8.6) and those that did not (mean MPS = 5.0, *p* = 0.0007). In summary, the MPS showed good interrater repeatability between expert and lay scorers, significant correlation with lameness at the walk and trot, and good predictive validity for longitudinal health outcomes, confirming its ability to identify horses with orthopaedic health problems.

## 1 Introduction

Musculoskeletal disease is the leading cause of chronic pain in horses and humans alike (1–8). In equine veterinary practice, lameness due to musculoskeletal pain ranks as the most prevalent diagnosis (1–4, 9–12). Already, in 4- to 5-year-old riding horses, 24% demonstrated moderate to severe orthopaedic clinical findings (12), emphasizing the widespread nature of the problem. The prevalence further increases in older horses, with 51% of horses above 15 years and 77% of horses aged 30 years and older exhibiting lameness, which is strongly associated with pain experienced at rest (3–5, 11, 13).

Despite their high prevalence, musculoskeletal diseases are frequently overlooked as a source of suffering and, as a result, receive inadequate treatment (2–4, 14–16). Indeed, owners reported lameness in only 16% of horses compared with the 77% diagnosed by veterinarians in the same cohort (11). Similarly, in two other groups of horses in training that were perceived to be sound by their owners, 72.5% and 74% showed movement asymmetry during objective lameness evaluation (17, 18). The owners’ low perception of musculoskeletal pain compared with the expert diagnoses is concerning from both a veterinary and welfare perspective. It further compounds the undertreatment of pain also observed in older humans that is associated with the erroneous but widespread societal belief that pain is a natural part of ageing and inevitable in later life (15, 19).

Due to the subjective nature of pain, *gold standard* pain assessment tools in human medicine rely on self-reporting, as direct measurement of individual experiences is not feasible (20, 21). For patients unable to communicate in ways easily understood by their caregivers, such as non-verbal human patients and animals, pain assessment depends on physiological and behavioural indicators (21–27). However, physiologic indicators, including changes in heart and respiratory rate, lack the sensitivity and specificity needed for reliable pain detection and discrimination from other sources of distress (28, 29). Although these indicators are commonly used to indicate the presence of pain, little empirical evidence exists to support this practice, as the correlation of vital sign changes with self-reports is weak, and the absence of changes in vital signs does not necessarily mean the absence of pain (28, 29).

By contrast, research has shown a strong correlation between pain behaviours and patients’ verbal pain reports, though external observers tend to underestimate pain intensity (30, 31). Consequently, non-verbal pain behaviours, such as facial expressions, lameness, and guarding, have become integral to pain assessments (22, 24, 27–42). Especially facial expressions, which have been demonstrated to encode both the sensory and affective components of pain, are commonly used to recognize and quantify pain in human and veterinary patients who are unable to verbalize (13, 26, 27, 37–50). Postural and gait adaptations that reduce the load on painful tissue to prevent or alleviate pain and protect from further injury (51) are also strongly associated with orthopaedic pain in humans and horses alike (35, 39, 40). However, despite evidence that guarding and posture may be more indicative of musculoskeletal pain than facial expressions (35, 39, 40), body cues are not routinely included in pain assessment.

As some behavioural changes associated with chronic pain may develop gradually and be subtle, making them most easily detected by someone familiar with the animal and its behaviour before and after the onset of pain (26, 52), the inclusion of caretaker assessments can add important cues to facilitate identification of equine pain. Regular pain assessment by caretakers is also essential to optimize treatment, as chronic musculoskeletal conditions typically require prolonged and often life-long palliative treatment and therapy adjustments to address acute flares and fluctuations in pain intensity while minimizing side effects. Hence, there is a clear need for a pain assessment tool that horse owners and veterinarians alike can use to facilitate the identification of pain, communication between veterinarians and clients, and the evaluation of the effectiveness of pain management interventions. This pain scoring system should be based on objective measures that are sensitive and specific to pain and minimize the potential for observer bias and misinterpretation (20, 23). Keeping in mind horses’ instinctive tendency to exhibit little indication of pain in the presence of potential predators, such as humans, and to reduce or relieve pain behaviour even during caretaker visits (46), the pain assessment tool should also be applicable remotely using video surveillance or recordings.

Therefore, this study aims to establish, refine, and validate an orthopaedic pain scale that is easy and fast to use by horse caretakers and veterinarians alike, and can also be used to score pain on videos to minimize observer interference with pain behaviour. Based on recent scientific evidence, the newly developed equine musculoskeletal pain scale (MPS) incorporates components of the equine pain face (27, 38), posture, head–neck position, weight-bearing, and weight shifting to assess orthopaedic pain in horses (33, 36, 39, 40, 44, 47, 53).

## 2 Materials and methods

This prospective, observational cohort study was designed to refine and validate the newly developed MPS, a tool based on components of the equine pain face (38), recent scientific advances demonstrating the importance of posture, weight-bearing, and head position for chronic pain behaviour (5, 13, 33, 36, 40, 44–47, 53–57), and clinical observations in patients suffering from chronic orthopaedic pain. During scale development and refinement, a panel of six experts (three equine veterinarians and three experienced equestrians) assessed content validity and comprehensibility through iterative evaluations of item relevance, comprehensiveness, and clarity (see Sections 2.3 and 2.5) (58–63). The size of the expert panel was based on previous studies establishing a minimum of four to five experts to be adequate for content validation (63, 64). In addition, item relevance was assessed by calculating the correlation between each item and the total MPS and by an item–total correlation (see Sections 2.3 and 2.7). To evaluate the influence of different sources of variability on the MPSs, reliability was determined by calculating the interrater variability in relation to the horses’ variability and the total variability in a mixed model (see Sections 2.3, 2.5, and 2.7) (58–63). The MPS’s construct validity was assessed by calculating the correlation of the MPS with subjective lameness scores at the walk and trot and the objective lameness data (see Section 2.4) (58–63). The scale's criterion validity to predict longitudinal health outcomes was evaluated by comparing the MPS score of horses that required veterinary intervention in the subsequent months with those that did not need medical treatment (see Section 2.4) (58–63). Lastly, the scale's responsiveness was assessed by comparing the MPSs of horses that received analgesia before and after treatment (58–63).

### 2.1 Horses

A total of 154 horses living at an equine sanctuary were included in this study. The horses were maintained in their familiar environment and husbandry conditions, and neither the horses’ housing, turn-out, or feeding regime nor any other management factors or veterinary treatments were affected by the study or changed for study purposes.

Before inclusion in the study, all horses underwent an in-depth physical exam. The horses suffering from non-orthopaedic causes of pain or cardiovascular (e.g., ventricular tachycardia) or gastrointestinal (e.g., delayed gastric emptying) disease were excluded from the study.

### 2.2 Horse examination and assessment parameters

All the horses were examined by the same veterinarian and received a complete clinical and orthopaedic exam in addition to an MPS. To complete the MPS, the horses were discreetly observed and videotaped from a suitable distance (5–10 m), displaying no awareness or curiosity towards the observer, while at rest in their box or enclosure (paddock or pasture). The MPS uses an ordinal scale to measure demeanour (13, 25, 26, 40, 44–47, 49, 65–67), pain face (22, 27, 38, 68, 69), weight shifting, weight-bearing, head–neck posture, limb posture (25, 33, 36, 44, 47, 53, 56, 57), and lameness that is evident while observing the horse from a distance in its enclosure (Supplementary Figure S1). Examinations involving direct interaction with the horses were conducted only after all initial distant observations were finished. This approach was taken to minimize any potential influence on the horses’ behaviour and any biasing of the MPS results by the examination process.

The orthopaedic exam included a subjective lameness evaluation grading the lameness at the walk and trot separately on a scale from 0 (sound) to 5 (non-weight-bearing) (70). The horses that were unable to trot because of severe lameness were assigned a score of 5 for the lameness at the trot. Horses with a lameness score >2 at the walk and ≥3 at the trot were considered moderately to severely lame.

In addition, the lameness was assessed objectively in 110 horses (71.4%) using a commercially available multi-sensor inertial gait analysis system (Lameness Locator®, Equinosis, USA) that has been validated to detect and quantify equine lameness (71–76). The horses were considered lame with a Q-score, a metric quantifying movement asymmetry amplitude, >8.5 mm and moderately to severely lame with a Q-score >30 mm (74–80).

### 2.3 Musculoskeletal pain scale—descriptive statistics

To characterize the MPS and its items, a correlation matrix was calculated among pairs of scores of items and the total using the non-parametric Spearman and the parametric Pearson correlations. In addition, a principal component analysis was calculated to further characterize the relationships among items. Both methods allow evaluation of which variables contribute independently or jointly to the total MPS.

### 2.4 Musculoskeletal pain scale—validity and predictive performance

The primary measures for assessing the validity of the MPS were the subjective and objective lameness scores. The correlations between the MPS with subjective lameness at the walk and trot and the objective lameness data were calculated using moderate to severe lameness as an indicator of pain.

To assess the MPS’s predictive performance for longitudinal health outcomes, the MPSs of horses that required veterinary intervention [analgesia (firocoxib or phenylbutazone) or euthanasia for pain that was unresponsive to treatment] in the months following the exam were compared retrospectively with those that did not need medical treatment. The need for veterinary intervention was determined by the nursing and veterinary staff of the sanctuary based on their independent assessment of the horses’ health and pain status, thereby providing an outcome variable independent from the study.

### 2.5 Musculoskeletal pain scale—interrater agreement and refinement

To ensure the inclusion of horses representing the entire spectrum of pain grades in the interrater agreement analysis, the horses were considered pain-free if their MPS was ≤3 (*n* = 64), mildly painful if their MPS was between 4 and ≤8 (*n* = 69), and moderately to severely painful if their MPS was ≥9 (*n* = 20). A subset of 30 horses, 10 of each pain group, was randomly selected using the GraphPad® random selection tool (https://www.graphpad.com/quickcalcs/randomselect2/). To determine the interrater agreement, six additional raters, three equine veterinary practitioners and three experienced equestrians, completed the MPSs for these 30 horses. The six additional raters were blinded to the horses' medical history, pain group, and exam results and completed the MPS based solely on anonymized videos obtained during the exam. Interrater reliability was assessed using intraclass correlation (ICC) analysis.

**Figure 1:**
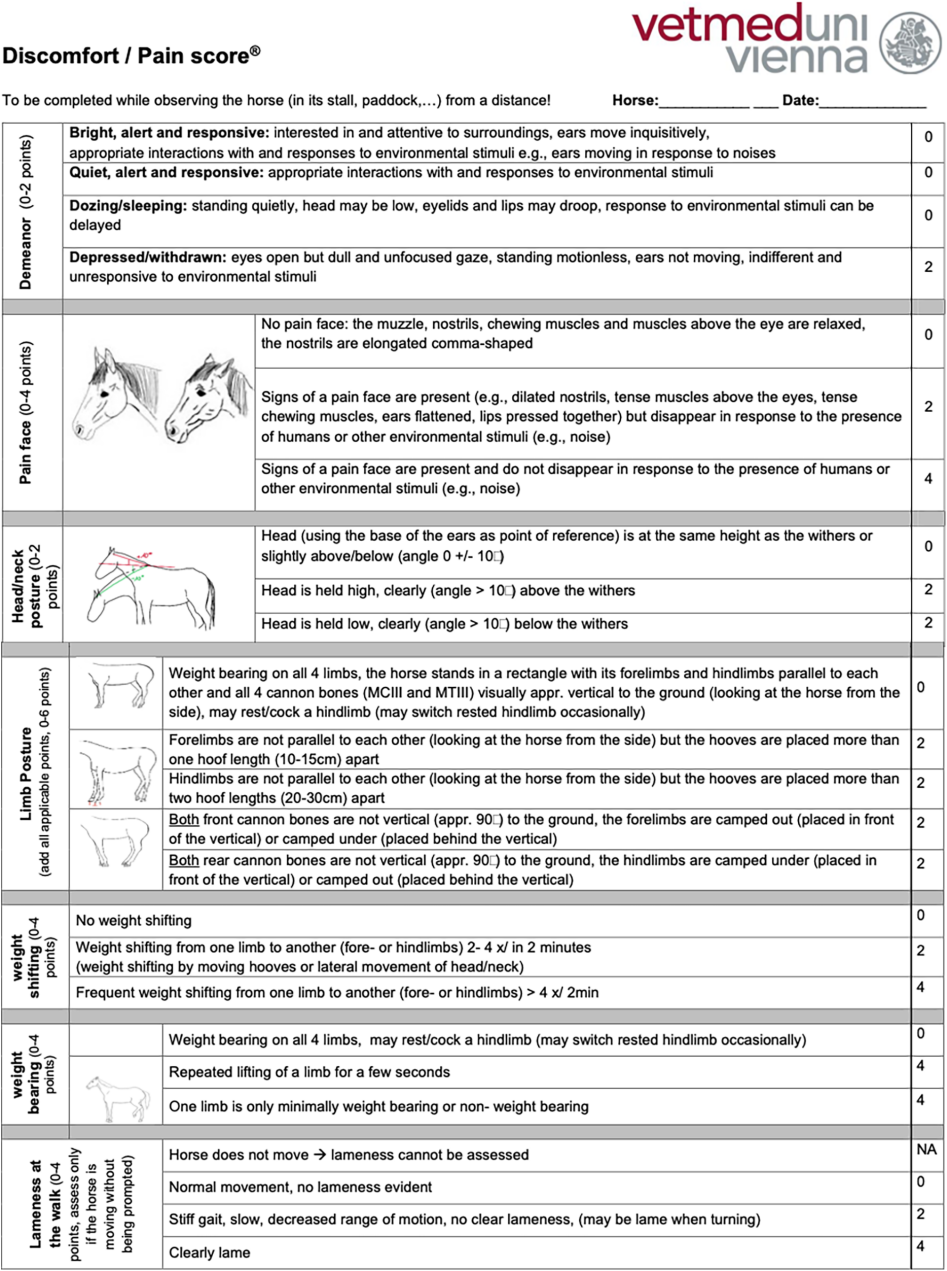
Refined musculoskeletal pain scale.

Based on the interrater agreement and their feedback regarding the clarity of the item descriptions and the scoring process, the MPS was refined to optimize the discriminative power of the items to ensure unequivocal definitions of each item to limit the potential for misinterpretation and to shorten the time required to complete the MPS to enhance its clinical and research utility. An iterative process was used in tool refinement, considering improvements achieved (content, construct, and criterion validity, comprehensiveness, comprehensibility, reliability, interrater agreement) compared with the original tool when replacing existing items or adding items. Item redundancy was investigated using correlation and principal component analysis. The refined MPS measures seven items on an ordinal scale and can accumulate a maximum score of 26 points, 2 for demeanour, 4 for a pain face, 2 for head–neck posture, 4 for weight shifting, 6 for limb posture, 4 for weight-bearing, and 4 for lameness that is evident while observing the horse from a distance in its enclosure (English version: Figure 1, German version: Supplementary Figure S2). The refined tool was tested in a new randomly selected subset of 30 horses representing the three pain groups (*n* = 10 per group) to assess the interrater agreement using ICC analysis and with a mixed model with rater-ID and horse-ID as random variables (see also Section 2.7). For the ICC, interrater agreement was considered to be very good (for scores 0.81–1.0), good (0.61–0.80), moderate (0.41–0.60), reasonable (0.21–0.4), or poor (<0.2) (81). Based on the excellent interrater agreement for the MPS’s lameness item established in the first validation step (ICC score 0.83), only observers one and two rated the lameness item, which had not been changed during the refinement process, as part of the last iteration of the MPS.

### 2.6 Refined musculoskeletal pain scale—validity and discriminative power

To assess the validity and discriminative power of the refined MPS, the refined MPS was completed for 60 video-recorded behavioural observations that had not been included in the first validation step (see Section 2.4). The construct validity was determined by correlation analysis of the MPS with the subjective lameness at the walk and the trot. The refined MPS’s criterion validity to predict longitudinal health outcomes was calculated by comparing the MPSs of the horses that required veterinary intervention with those that did not, using a Mann–Whitney *U* test. The MPS’s responsiveness, its ability to discriminate between before and after treatment, was assessed by comparing the MPSs of the horses receiving analgesia before and after intervention.

Receiver operating characteristic (ROC) analysis was utilized to evaluate the global performance of the MPS in discriminating between lame and sound horses and between horses that needed veterinary intervention and those that did not. In addition, the ROC was used to determine cut-off values that minimize misclassification errors (82–84). The optimal cut-off for discriminating between horses suffering from pain and those without any pain was identified as the value where the sum of the sensitivity [=probability of a positive test outcome in a horse that is in pain (true-positive)] and specificity [=probability of a negative test outcome in a pain-free horse (true-negative)] was maximized. If two cut-off values yielded similar sums of sensitivity and specificity, the cut-off with the higher sensitivity was chosen to maximize the likelihood of identifying horses suffering from pain for further diagnostics and therapy if required. Since the MPS item lameness can only be assessed if the horse is moving in its stall or enclosure without being prompted, scoring this item may not always be possible, which could result in a lower maximum MPS. Therefore, we determined the cut-off value for the refined MPS, both including and excluding the lameness item.

### 2.7 Statistical analyses

Statistical analyses were carried out using GraphPad Prism (version 10.0.2, GraphPad Software LLC, Boston, MA, USA), NCSS 2020 Statistical Software (NCSS, LLC. Kaysville, UT, USA), and the “R” statistical programming language (R Foundation for Statistical Computing, Vienna, Austria, https://www.R-project.org/) (85). The D'Agostino–Pearson, Shapiro–Wilk, Anderson–Darling, and Kolmogorov–Smirnov tests were computed to assess whether data were normally distributed. The *t*-test, ANOVA, and Pearson's correlation test were used for normally distributed parameters, whereas for parameters that were not normally distributed, the Mann–Whitney *U*, Kruskal–Wallis, and the Spearman correlation tests were calculated. A principal component analysis was performed to describe the relationships among the MPS items. For the item–total correlation, the value of the focal item was correlated with the MPS minus the value of the focal item. Furthermore, a mixed model with horse and rater as random effects was computed to evaluate the relative contribution of each to the total variation. The *p*-values < 0.05 were considered statistically significant. For correlation analysis, correlation coefficients |*r*| < 0.3, 0.3 ≤ |*r*| ≤ 0.8, and |*r*| > 0.8 were considered to indicate weak, significant, and strong correlations, respectively. For ROC analysis, the concordance statistic (*c*-statistic, equivalent to the area under the ROC curve) represents the probability that a randomly selected patient will have a higher test result than a randomly selected control. It is utilized as a measure of the global accuracy of a diagnostic test and is considered to indicate low, moderate, and high test accuracy at values of 0.5 < *c* ≤ 0.7, 0.7 < *c* ≤ 0.9, and *c* > 0.9, respectively (82–84).

## 3 Results

### 3.1 Horses

The 154 horses included 67 warmbloods, 25 draft, 25 Arabian, 18 Haflinger horses, and 19 horses of other breeds. The horses were 2–32 years old [mean: 20 years, SD: 6.4 years, median: 21 years, interquartile range (IQR): 16–26 years]. Of the 154 horses, 128 (83%) suffered from chronic orthopaedic disease, such as osteoarthritis (*n* = 74/154, 48%), tendinopathy (*n* = 29/154, 19%), or laminitis (*n* = 25/154, 16%) according to their medical records.

### 3.2 Horse examination and assessment parameters

The 154 horses had a mean original MPS of 4.8 (SD: 3.0, range: 0–14, median: 4.0, IQR: 2.8–7.0). The 11 horses unable to trot were scored 5 for the subjective lameness exam at the trot and assigned a Q-score of 115 (10% higher than the maximum measured Q-score of 104.7). The mean subjective lameness score (maximum of the four limbs, scale of 0–5) at the walk was 1.6 (SD: 1.2, range: 0–5, median: 2.0, IQR: 0.75–2.0) and at the trot, 2.2 (SD: 1.1, range: 0–5, median: 2.0, IQR: 2.0–3.0). The mean Q-score of the objective lameness exam was 28 mm (SD: 35 mm, range: 0.0–104.7 mm, median: 13 mm, IQR: 8.4–23 mm).

The cohort of 60 horses used to validate the refined MPS had a mean MPS of 6.9 (SD: 4.3, range: 0.0–18.0, median: 6.0, IQR: 4.0–10.0) and a mean lameness of 2.1 at the walk (SD: 1.2, range: 0.0–4.0, median: 2.0, IQR: 1.0–3.0) and 2.7 at the trot (SD: 1.6, range: 0.0–5.0, median: 3.0, IQR: 2.0–3.0). One horse was excluded from the lameness exam owing to chronic ataxia.

### 3.3 Musculoskeletal pain scale—descriptive statistics

**Figure 2:**
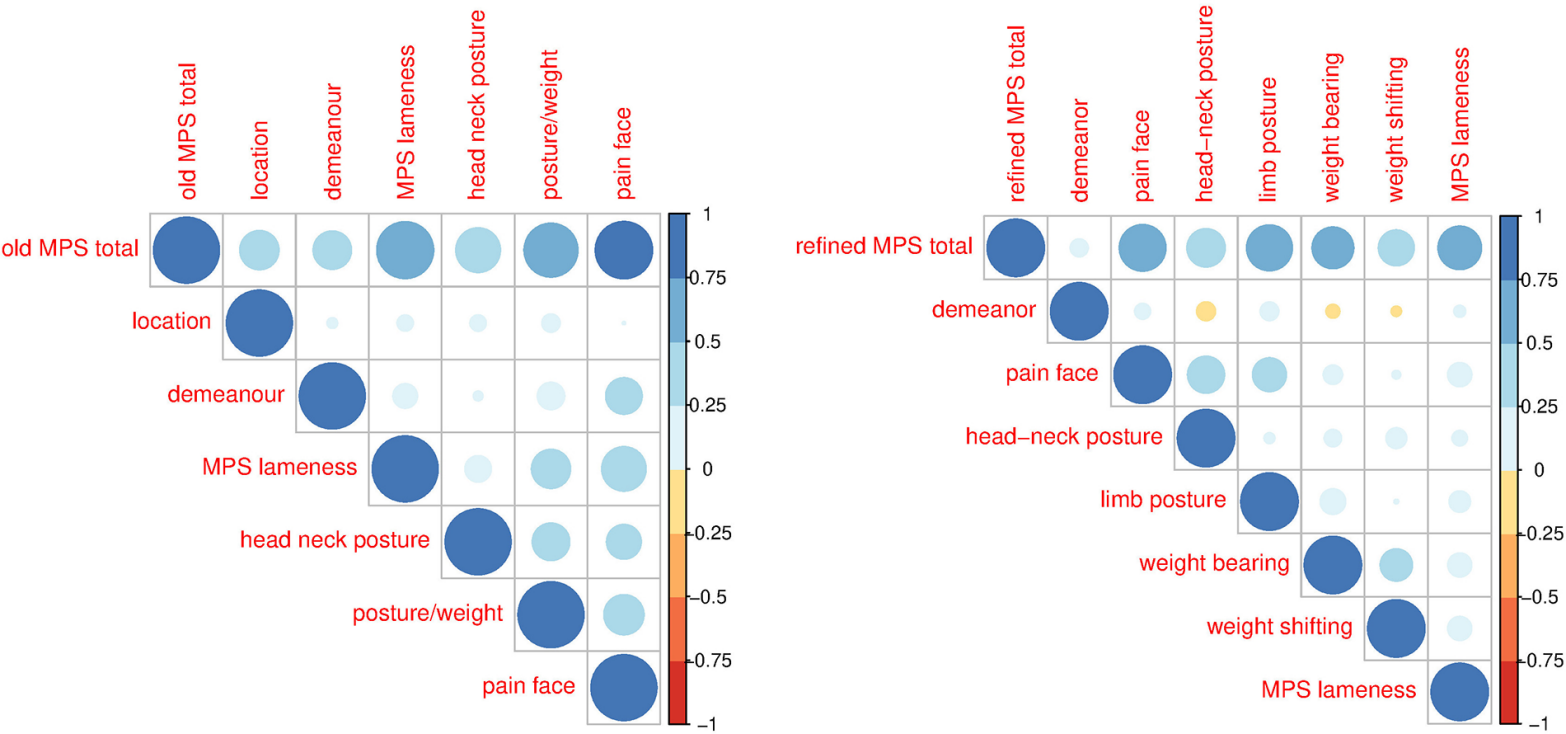
Pearson correlations for the original (left) and refined (right) MPS for the total and the various sub-items of the MPS. The colours and sizes of the balls indicate the strength of the correlation.

The correlations among items and their contribution to the total score were assessed using correlation analysis and principal component analysis. The correlation analysis using the non-parametric Spearman and the parametric Pearson correlations showed negligible or positive correlations of varying strengths among the items of the original MPS (Figure 2). In the original MPS, the item “location in the box/enclosure” correlated little with the other items (*r* < 0.25) and had a correspondingly low item–total correlation of 0.092, while the other items correlated with a correlation coefficient between about 0.3–0.4 and had item–total correlations between 0.18 and 0.445. The correlation of the items with the total original MPS is also shown, from which it is evident that location, demeanour, and head–neck posture have a lower correlation than lameness, weight distribution, and pain face, which show pairwise correlations in the range 0.25–0.75. Using the non-parametric Spearman or the parametric Pearson correlation produced qualitatively and quantitatively similar patterns. As expected from the correlation analysis, all subitems, except demeanour and location, contribute positively to the first principal component, especially lameness and pain face, which load highly (Table 1), while they load with opposite signs on the second principal component. All other items generally dominate one further principal component.

**Table 1:** The proportion of the principal components indicating their relative importance are given in the first row. The loadings of the items, reflecting their contributions to the principal components, are given in the following for the original (A) and refined (B) MPS. Values below 0.1 are not reported, i.e., left blank.

A							
Component		1	2	3	4	5	6
Location			0.592	0.767	0.243		
Demeanour						0.109	−0.990
Pain face		0.604	−0.605	0.299	0.404		
Head–neck posture		0.112		0.139	−0.164	−0.963	
Limb posture/weight-bearing/shifting		0.331		0.293	−0.864	0.229	
Lameness		0.712	0.521	−0.465			
Proportion of variance		0.483	0.171	0.159	0.119	0.049	0.019
B							
Component	1	2	3	4	5	6	7
Demeanour							0.998
Pain face	0.460		−0.747		0.379	0.281	
Head–neck posture	0.127		−0.479	−0.140	−0.670	−0.529	
Limb posture	0.767	0.467	0.390		−0.176		
Weight-bearing	0.204	−0.133	0.108	−0.845	0.371	−0.276	
Weight shifting		−0.166		−0.416	−0.489	0.746	
Lameness	0.371	−0.856	0.221	0.276			
Proportion of variance	0.369	0.218	0.163	0.124	0.0653	0.0549	0.006

### 3.4 Musculoskeletal pain scale—validity and predictive performance

The MPS correlated significantly with the subjective lameness score at the walk (Spearman *r* = 0.51, *p* < 0.0001) and the trot (Spearman *r* = 0.45, *p* < 0.0001) and the objective lameness measurements (Spearman *r* = 0.37, *p* = 0.0001, *p* ≤ 0.0001).

The MPS was significantly different (difference between means: 2.1 ± 0.5 SEM, *p* < 0.0001) between horses that needed veterinary intervention (mean MPS: 6.2, *n* = 49) and those that did not (mean MPS: 4.1, *n* = 105). The MPS was also significantly different (difference between means: 4.3 ± 0.5 SEM, *p* < 0.0001) between horses that were clearly lame at the walk (lameness ≥3, mean MPS: 8.3, *n* = 29) and those that were not or only mildly lame (grade 0–2, mean MPS: 4.0, *n* = 125). Similarly, the MPS was significantly different (difference between means: 2.9 ± 0.5 SEM, *p* < 0.0001) between horses that were clearly lame at the trot (lameness ≥3, mean MPS: 6.8, *n* = 45) and those that were not or only mildly lame (grade 0–2, mean MPS: 3.9, *n* = 109). Furthermore, the MPS was significantly different between objectively measured lameness scores, specifically between no lameness (Q-score ≤ 8.5, mean MPS: 3.5) and moderate to severe lameness (Q-score > 30, mean MPS: 7.9, *p* < 0.0001), and between mild (Q-scores 8.5–30, mean MPS: 4.1) and moderate to severe lameness (*p* < 0.0001), but not between mild and no lameness.

### 3.5 Musculoskeletal pain scale—interrater agreement and refinement

During the refinement process, the item “location in the box/enclosure” was dropped as it was not consistently possible to reliably rate the location on videos, and the MPS was intended to allow for remote scoring to avoid the confounding effect of rater presence on horses’ behaviour. The original item, scoring limb posture, weight-bearing, and weight shifting together were divided into three items in the refined tool. The definition of the other items was optimized based on feedback from the raters and interrater agreement to optimize clarity and minimize the potential for misinterpretation. The time required to complete pain assessment was reduced from 7 min with the original MPS to 2 min with the refined MPS, hence enhancing its user-friendliness and corresponding clinical and research utility.

Correlations among the items of the refined MPSs (Figure 2) were lower than for the original MPS, indicating lower item redundancy; demeanour correlated little with the other items (*r* < 0.25); pain face correlated moderately with head–neck and limb posture, and weight-bearing correlated with weight shifting, all with a correlation coefficient between 0.3 and 0.4. The correlation of the items with the total refined MPS is below 0.1 for demeanour and between 0.25 and 0.6 for the other items. The item–total correlation for demeanour was only 0.053, that of the rest of the items between 0.19 (weight shifting) and 0.464 (pain face). Using the non-parametric Spearman or the parametric Pearson correlation produced qualitatively and quantitatively similar patterns. The first principal component has positive loadings for all items of the refined score except demeanour and weight shifting (Table 1). While the item demeanour dominates one principal component of the refined MPS, the loadings of all other items are generally more dispersed over the various other components than for the original MPS. The residual variance can be attributed to the variation in the video quality and hence as technical variance.

The intraclass correlation increased from 0.77 [95% confidence interval (CI): 0.62–0.88] for the original MPS to 0.88 (95% CI: 0.80–0–94) for the refined MPS tool (Supplementary Table S1).

A mixed model analysis evaluated the variance due to variability among horses, among raters, and the residual variance (Table 2). The variability among horses is more than five times higher than that among raters for the total scores as well as lameness, head–neck posture, and limb posture, indicating that these variables can be reliably scored by the different raters. The ratio is less favourable for pain face, weight-bearing, and weight shifting. The residual variance can be attributed to the variation in the video quality and hence as technical variance.

**Table 2:** Mixed model analysis of the total refined MPS and its various subitems.

Variance component	Horse-ID	Rater-ID	Residual
Refined MPS	6.935	0.820	5.313
Demeanour	0.020	0.003	0.158
Pain face	0.555	0.004	1.228
Head–neck posture	0.301	0.003	0.502
Limb posture	1.116	0.482	1.260
Weight-bearing	0.771	0.331	1.429
Weight shifting	0.523	0.000	0.333
Lameness	-	-	-

### 3.6 Refined musculoskeletal pain scale—validity and discriminative power

The refined MPS was validated by its significant correlation with the subjective lameness score in walk (Spearman *r* = 0.44, *p* = 0.001) and trot (*r* = 0.5, *p* < 0.0001).

**Figure 3:**
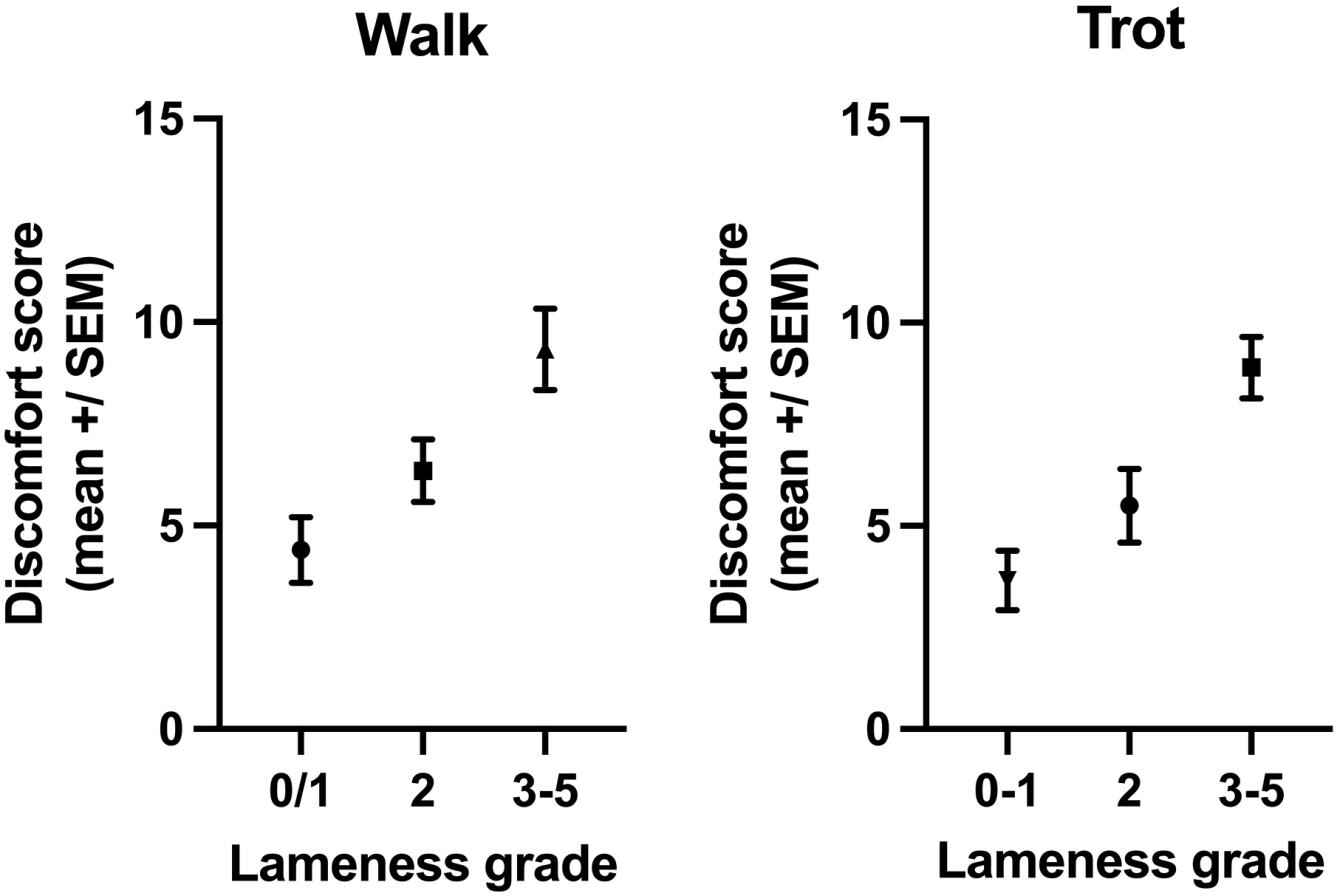
The MPSs of horses with a lameness ≥3 at the walk (*p* = 0.0009) or trot (*p* < 0.0001) were significantly higher than the MPSs of horses with no or mild lameness (lameness 0–2).

The MPS was significantly different (difference between means: 3.8 ± 1.1 SEM, *p* = 0.0009) between horses that were clearly lame in walk (lameness ≥3, mean MPS: 9.3, *n* = 21) and those that were not or only mildly lame (grade 0–2, mean MPS: 5.6, *n* = 38, Figure 3). Furthermore, the MPS was significantly different (difference between means: 4.2 ± 0.99 SEM, *p* < 0.0001) between horses that were clearly lame at the trot (lameness ≥3, mean MPS: 8.9, *n* = 31) and those that were not or only mildly lame (grade 0–2, mean MPS: 4.7, *n* = 28, Figure 3).

**Figure 4:**
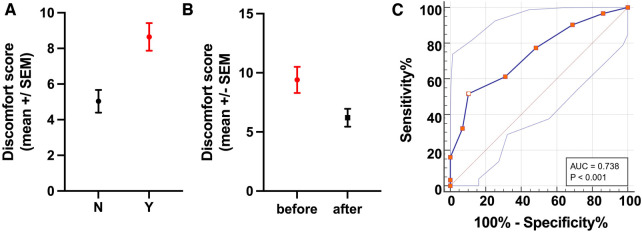
Predicative performance and discriminative power for longitudinal health outcomes. (**A**) The refined MPS showed good discriminative power for longitudinal health outcomes as demonstrated by the significant difference (*p* = 0.0007) in the MPSs of horses that needed veterinary interventions (Y, red) compared with those that did not (N, black). (**B**) Evaluation of the MPS responsiveness showed a significant difference (*p* = 0.0168) in the MPSs of horses receiving analgesia before and after treatment. (**C**) The ROC curve plots 100% − specificity% vs. sensitivity% for each MPS value. Using a cut-off for the MPS >8 (shown as a white dot with red border) yields a sensitivity of 51.61% and a specificity of 89.66% (Youden index: 0.41) for identifying horses in need of veterinary intervention (analgesia or euthanasia for unrelenting pain). Thus, horses with an MPS of 8 or above are likely to have a painful condition and should be further examined.

The refined MPS was also significantly different (difference between means: 3.6 ± 1.0 SEM, *p* = 0.0007) between horses that needed veterinary intervention (mean MPS: 8.6, *n* = 31) and those that did not (mean MPS: 5.0, *n* = 29, Figure 4), hence establishing the predictive performance of the MPS for longitudinal health outcomes.

Lastly, the MPS was significantly different (difference between means: 3.2 ± 1.093 SEM, *p* = 0.0168) in horses receiving analgesia between before and after treatment, confirming its responsiveness (Figure 4).

ROC analysis showed the refined MPS to be moderately accurate in discriminating between horses that needed veterinary intervention and those that did not, with a c-statistic of 0.74 (standard error: 0.064, *p* = 0.0002). The ROC analysis yielded several possible MPS cut-off values with corresponding trade-offs in sensitivity and specificity (Table 3, Figure 4, Supplementary Table S2), with an MPS cut-off >8 providing the best overall combination of sensitivity (51.61%, 95% CI: 34.84%–68.03%) and specificity (89.66%, 95% CI: 73.61%–96.42%) with a Youden index (=specificity plus sensitivity minus one) of 0.41, indicating that horses with an MPS of 8 or greater have a high probability of a painful condition and therefore should be further examined.

**Table 3:** Sensitivity and specificity plus 95% CI calculated by ROC analysis for each MPS cut-off value for identifying horses needing veterinary intervention.

Cut-off	Sensitivity (%)	95% CI	Specificity (%)	95% CI	Youden index	Likelihood ratio
Differentiation between horses that needed medical intervention and those that did not						
>0	96.77	83.81%–99.83%	13.79	5.5%–30.56%	0.11	1.123
>2.0	90.37	75.10%–96.65%	31.03	17.28%–49.23%	0.21	1.31
>4.0	77.42	60.19%–88.60%	51.72	34.43%–68.61%	0.29	1.6
>6.0	61.29	43.82%–76.27%	68.97	50.77%–82.72%	0.3	1.975
>8.0	51.61	34.84%–68.03%	89.66	73.61%–96.42%	**0.41**	4.989
>10.0	32.26	18.57%–49.86%	93.1	78.04%–98.77%	0.25	4.677
>12.0	16.13	7.093%–32.63%	100.0	88.30%–100.0%	0.16	
>16.0	3.226	0.166%–16.19%	100.0	88.30%–100.0%	0.03	

The best Youden index (the sum of sensitivity and specificity − 1) is indicated in bold.

Without the item lameness, ROC analysis (*c*-statistic: 0.65, standard error: 0.071, *p* = 0.034) showed that an MPS cut-off of >4 (Youden index of 0.264) yielded the best combination of sensitivity (67.74%, 95% CI: 51.6%–74.2%) and specificity (58.62%, 95% CI: 48.4%–75.5%, Supplementary Figure S2). The difference in the MPSs (difference between means: 2.0 ± 0.9) between horses that needed veterinary intervention (mean: 6.3) and those that did not (mean: 4.3) remained statistically significant also without the lameness item (*p* = 0.0331, Supplementary Figure S2).

## 4 Discussion

The high prevalence and impact of chronic musculoskeletal conditions and the poor recognition of lameness and the associated pain necessitate the inclusion of pain as a fourth vital sign in the routine evaluation of all horses to facilitate appropriate treatment and improve equine welfare (52, 86, 87). Regular pain assessment using a reliable, valid, and clinically useful tool would enable the identification of pain, timely interventions, monitoring treatment effects, and facilitate communication among veterinarians and caretakers. This study established, refined, and validated a multidimensional MPS and demonstrated its predictive performance for longitudinal health outcomes, discriminative power, and good interrater agreement between veterinary practitioners and equestrian raters. The MPS was validated as a measure for equestrians and veterinarians alike to assess horses for the presence of painful conditions and monitor the efficacy of treatment interventions, not just in a hospital setting but especially also in their home environment. The MPS is based on several pain behaviours, including features of the equine pain face, postural indicators, demeanour, and lameness to accommodate for individual variations in pain behaviour and differences in pain behaviours between acute and chronic pain (13, 26, 40, 41, 88–91).

Pain is a complex, uniquely individual, unpleasant experience associated with actual or potential tissue damage, encompassing both sensory (intensity) and affective (unpleasantness) components (92). The affective dimension of pain is associated with behavioural changes aimed at avoiding pain and minimizing injury (23, 54, 93). While acute pain is a protective response to noxious stimuli, chronic pain persists beyond the expected healing time and may be either a symptom of chronic peripheral disease, maladaptive nervous system dysfunction, or both (89, 90, 94–100). Acute pain tends to respond to anti-inflammatory pain relief. Decreasing the load on the affected area by postural adaptations and guarding during movement and at rest may reduce acute pain (13, 40, 41). By contrast, in chronic pain, due to the central sensitization that is often present, the degree, duration, and spatial extent of pain may be increased and distorted, leading to more widespread pain and multisite hyperalgesia and allodynia (13, 40, 86, 88, 89, 95, 101–103). Therefore, once central sensitization has occurred, pain perception may no longer reflect the presence, intensity, or duration of peripheral noxious inputs (103, 104). Accordingly, behavioural changes associated with chronic pain can vary greatly, necessitating a multidimensional pain assessment tool that includes the effect of pain on demeanour, functional assessments (lameness), and different pain behaviours (24, 41, 86, 89–91, 94–97, 99, 105, 106). Pain behaviours can be categorized into two overlapping groups: protective and communicative pain behaviours (106). Protective pain behaviours, such as postural adjustments and guarding, are often directly associated with the painful area (13, 40). By contrast, communicative pain behaviours, including facial expressions, are universal indicators of pain and mechanisms for communicating pain to conspecifics (13, 40).

Posture, the dynamic alignment and positioning of the body orchestrated by the neuromuscular system, is often mistakenly conflated with conformation, which pertains to the static skeletal architecture and body proportions (13, 33, 40, 45, 47, 48, 107, 108), confounding research into the complex interplay between posture and musculoskeletal pain. Posture facilitates efficient weight distribution across the musculoskeletal system, balancing the centre of gravity over the base of support to minimize energy expenditure and stress on anatomical structures (33, 109, 110). As symmetrical loading of the limbs provides the greatest biomechanical stability and hence requires the least corrective actions and energy to maintain balance (110), sound horses exhibit a symmetrical weight distribution, with approximately 60% of the weight borne by the forelimbs and 40%, by the hindlimbs (33, 111, 112). Conversely, horses afflicted with orthopaedic conditions may attempt to alleviate pain by shifting the weight away from the affected limb, effectively altering their centre of gravity (113). Weight-bearing and stance asymmetry may therefore signal pain relieved by adopting this posture (112). Similarly, an elevated neck posture has been identified as a potential indicator of underlying back disorders in horses (53, 56, 57, 107, 114, 115). Therefore, the MPS includes head–neck posture, limb posture, weight-bearing, and weight shifting as separate items to reflect the postural adaptations commonly observed in response to orthopaedic pain. We note that due to the complexity of pain, a single item may not correlate highly with other items or show a low item–total correlation, i.e., pain may represent more than one dimension. This may explain why the factor loadings are relatively low and the proportion of the variance explained by the principle components is rather even, especially with the refined MPS. In particular, pain face may integrate many aspects of pain, while other items may reflect a specific condition or individualized reaction.

The horses’ interaction with humans may also be variably affected by pain (25, 38, 44). Depending on the intensity of the noxious stimuli and the familiarity of the environment and observer, painful horses may either be reluctant to interact with humans or increase their contact-seeking behaviour (25, 38, 44). Conversely, the horses may reduce or relieve pain behaviour when people approach or interact (46), which can lead to underestimation of the pain and subsequent therapeutic deficits and welfare problems. Therefore, the MPS was designed to be applicable from a distance to avoid disrupting pain behaviours.

This study established the content, criterion, and construct validity of the MPS using an expert panel for content validation, correlation with lameness for criterion validation, and correlation with longitudinal health outcomes for construct validation. As criterion validation assesses how accurately a scale reflects the *gold standard* for measuring the same construct (63), the lacking *gold standard* or other previously validated method for measuring the individual experience of pain is one of the main limitations of this study, which uses lameness as an indicator of orthopaedic pain. Although lameness is a reliable indicator of pain, the absence of overt lameness does not exclude the possibility of pain. This limitation, the lacking gold standard and objective, of quantitative pain measurement also extends to the evaluation of the MPS’s construct validity, the assessment of its ability to discriminate between horses in pain and pain-free horses (63). This study uses the need for veterinary intervention, identified by the staff of the sanctuary, to assess the MPS’s construct validity. The inherent subjectivity of this assessment is however mitigated by the horses statistically significant reduction in pain in response to analgesia. However, multicentre studies using larger patient cohorts are needed to further evaluate the MPS’s utility to identify horses in pain in various husbandry and demographic settings.

While the MPS is a quantitative tool, it is crucial to recognize that pain expression does not directly correlate with the severity of tissue damage but reflects horses’ individual experience and personality and that many pain behaviours are part of the communication repertoire of healthy horses as well (26). However, the MPS correlates well with lameness at the walk and trot and showed very good predictive performance for longitudinal health outcomes and discriminative power in identifying lame horses and horses needing veterinary intervention. In this cohort, the maximum MPS was 18 (the horse was 3/5 lame at the walk and too lame to trot), the maximum score of 26 was not reached by any horse, possibly because no horse in this study suffered from severe pain. Based on the ROC analysis and the differences between horses exhibiting obvious lameness (≥3 on a scale of 0–5) or requiring veterinary intervention, as opposed to horses with minimal or no observable health concerns, horses with an MPS exceeding 8 (or 4 if the lameness item cannot be assessed) should undergo further examination to identify the underlying cause and determine if treatment is necessary.

## 5 Conclusions

In summary, the MPS showed good interrater repeatability between expert and lay scorers, significant correlation with lameness at the walk and trot, and good predictive validity for longitudinal health outcomes, confirming its ability to identify horses with musculoskeletal pain. Given the prevalence of chronic musculoskeletal conditions, the poor recognition of lameness, and the suffering caused by unrelieved pain, pain assessment should be included in all veterinary examinations, and caretakers should regularly evaluate their horse's pain status to facilitate timely therapeutic interventions. Routine pain assessment using a reliable and validated tool may help address the widespread problem of unrelieved pain already voiced by the philosopher Michel de Montaigne in 1589: “For heaven's sake, let medicine someday give me some good and perceptible relief and you will see how I shall cry out in good earnest: At last I yield to an efficient science.”

## Data Availability

The raw data supporting the conclusions of this article will be made available by the authors, without undue reservation.
