# Supplementary material for: Development, refinement, and validation of an equine musculoskeletal pain scale
Source: Front Pain Res (Lausanne). 2024 Jan 19;4:1292299. doi: 10.3389/fpain.2023.1292299 (PMC10837853; doi:10.3389/fpain.2023.1292299)
Supplement: Supplementary file 1 [file Datasheet1.pdf]

Development, refinement, and validation of an equine musculoskeletal pain scale

Supplementary figure 1: Original musculoskeletal pain scale

Discomfort / Pain score®

To be completed while observing the horse (in its stall, paddock,…) from a distance! **Horse:**___________ ___ **Date:**_____________

**Demeanor** (0-2 points)		**Bright, alert and responsive:** interested in and attentive to surroundings, ears move inquisitively, appropriate interactions with and responses to environmental stimuli e.g., ears moving in response to noises	0
		**Quiet, alert and responsive:** appropriate interactions with and responses to environmental stimuli	0
		**Dozing/sleeping:** standing quietly, head may be low, eyelids and lips may droop, response to environmental stimuli can be delayed	0
		**Depressed/withdrawn:** eyes open but dull and unfocused gaze, standing motionless, ears not moving, indifferent and unresponsive to environmental stimuli	2
**Location in the box/paddock** (0-3 points)		Box: Stands next to the stable door<br>Paddock: stands in company of other horses, no evident agonistic behaviour	0
		Box: Stands distant from the stable door but looks toward the door<br>Paddock: stands in company of other horses, withdraws when approached	1
		Box: Stands distant from the stable door and faces to the side<br>Paddock: stands in vicinity but not in company of other horses, withdraws when approached	2
		Box: faces to the back of the stable<br>Paddock: stands alone, withdraws when approached, agonistic behaviour	3
**Pain face** (0-4 points)	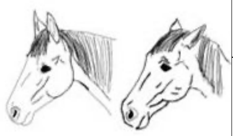	No pain face	0
		Signs of a pain face are present but disappear in response to the presence of humans or other environmental stimuli (e.g., noise)	2
		Signs of a pain face are permanently present	4
**Head/neck posture** (0-2 points)		Head (using the base of the ears as point of reference) is at the same height as the withers	0
		Head is held above or below the withers	1
		Head is held clearly above or below the withers	2
**Posture** (0-4 points)	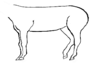	Weight bearing on all 4 limbs, the horse stands in a rectangle, may rest/cock a hindlimb (may switch rested hindlimb occasionally)	0
	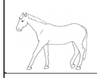	Uneven weight distribution, horse is not standing in a rectangle, grade 1: the hooves are places < 0.5 hoof lengths apart (1 point), grade 2: the hooves are placed one hoof length apart (2 points), grade 3: the hooves are placed more than one hoof length apart (3 points)	1-3
	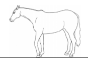	Weight shifting from one limb to another (fore- or hindlimbs), Repeated lifting of a limb<br>Grade 1: 1-2 x/5min (1 point), Grade 2: 3-5 x/5 min (2 points), Grade 3: > 5x/5min	1-3
	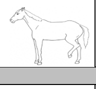	One limb is only minimally weight bearing or non- weight bearing, continuous weight shifting, abnormal body posture	2
**Lameness at the walk** (0-4 points, assess only if the horse is moving without being prompted)		Horse does not move → lameness cannot be assessed	NA
		Normal movement, no lameness evident	0
		Stiff gait, slow, decreased range of motion, no clear lameness, (may be lame when turning)	2
		Clearly lame	4

Supplementary figure 2: Refined musculoskeletal pain scale in German

Discomfort /Pain score®

Aus der Ferne zu beurteilen wenn das Pferd ungestört (in der Box, auf dem Paddock,…) ist! **Pferd:**____________ **Datum:**_____________

**Bewusstseinszustand** (0-2 Punkte)		**Wach:** steht ruhig oder frisst oder geht herum, Kopf bewegt sich, verfolgt/reagiert auf Geräusche, Augen offen, wacher Blick, beobachtet Umgebung, reges Ohrspiel mit Reaktion auf Geräusche	0
		**Dösen:** Ruhiges Stehen, kaum bzw. nur minimale Kopfbewegungen, Augen halbgeschlossen, „Blick ins Leere“, reagiert auf Geräusche (verzögert) mit Kopf und Ohrspiel,	0
		**Schlafen:** Ruhiges Stehen, minimale Kopfbewegungen (sinkt langsam ab), Augen halbgeschlossen, reagiert auf Geräusche (sehr verzögert) mit Kopf und Ohrspiel, streckt sich, verlagert Gewicht und geht wieder in Ruheposition	0
		**In sich gekehrt:** Ruhiges stehen, keine Kopfbewegungen, keine Reaktion auf Umweltgeräusche oder Bewegungen, Augen offen bis halbgeschlossen, nach „Innen“ gerichteter, (glanzloser) Blick, Ohren seitlich hängend oder rückwärtsgerichtet, kein oder sehr verzögertes Ohrspiel	2
**Schmerzgesicht** (0-4 Punkte)	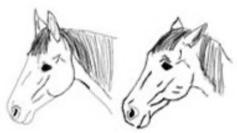	Keine Anzeichen von Schmerzgesicht: die Nase, die Nasenlöcher, die Kaumuskeln und die Muskeln über dem Auge sind entspannt, die Nasenlöcher sind langgestreckt und kommaförmig	0
		Anzeichen eines Schmerzgesichts sind vorhanden (z. B. geweitete Nasenlöcher, angespannte Muskeln über den Augen, angespannte Kaumuskeln, abgeflachte Ohren, zusammengepresste Lippen), verschwinden bei Anwesenheit von Menschen oder Umweltreizen (z. B. Lärm)	2
		Deutliches Schmerzgesicht, bleibt dauerhaft	4
**Relation Kopf-Widerrist** (0-2 Punkte)	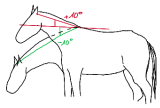	Kopf (Referenz Ohrbasis) auf Höhe des Widerrists oder knapp (Winkel 0 +/- 10□) darüber/darunter	0
		Kopf hoch, deutlich (Winkel > 10□) oberhalb des Widerrist (Referenz Ohrbasis)	2
		Kopf tief, deutlich (Winkel > 10□) unter Widerrist (Referenz Ohrbasis)	2
**Gliedmaßenstellung in Ruhe** (alle zutreffenden Punkte zusammenzählen, 0-6 Punkte)	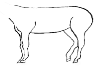	Gleichmäßige Gewichtsverteilung, Hufe stehen im Rechteck mit Vorderhufen und Hinterhufen jeweils parallel zueinander (von der Seite betrachtet) und den Rohrbeinen (MCIII und MTIII) im Lot (90□Winkel zum Boden), evt. Ruhestellung mit Schildern der Hinterextremitäten (wechselt gelegentlich)	0
	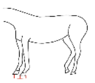	Vorderhufe stehen - von der Seite betrachtet - nicht parallel zueinander, sondern mehr als eine Huflänge (10-15 cm) versetzt	2
		Hinterhufe stehen - von der Seite betrachtet - nicht parallel zueinander, sondern mehr als 2 Huflängen (20-30 cm) versetzt	2
	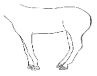	<u>Beide</u> Vordergliedmaßen stehen - von der Seite betrachtet - nicht im Lot (MCIII (Rohrbein) nicht im 90□Winkel zum Boden) sondern vorständig oder rückständig/unterständig	2
		<u>Beide</u> Hintergliedmaßen stehen - von der Seite betrachtet - nicht im Lot (MTIII (Rohrbein) nicht im 90□Winkel zum Boden) sondern vorständig/unterständig oder rückständig	2
**Gewichtsver-lagerung** (0-4 Punkte)		Keine Gewichtsverlagerung / weight shifting sichtbar	0
		Gewichtsverlagern von einer Extremität zur anderen (vorne oder hinten): 2- 4 x/ in 2 Minuten (Gewichtsverlagerung durch Huf umstellen und/oder Seitbewegung von Hals/Kopf)	2
		Häufiges Gewichtsverlagern von einer Extremität auf die andere (vorne oder hinten) >4x / 2 Minuten	4
**Belastung in Ruhe** (0-4 Punkte)		Gleichmäßige Gewichtsverteilung, evt. Ruhestellung mit Schildern der Hinterextremitäten (wechselt gelegentlich)	0
	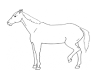	Wiederholtes Beugen oder Hochheben einer Extremität mit kurzer Entlastung für wenige Sekunden	2
		Ständige Entlastungshaltung einer Gliedmaße (vorne oder hinten), wodurch die Gewichtsbelastung dieser Gliedmaße sichtbar reduziert wird	4
**Lahmheit im Schritt** (0-4 Punkte, nur zu beurteilen falls das Pferd sich ohne Aufforderung bewegt)		Pferd bewegt sich nicht → daher ist die Lahmheit nicht beurteilbar	NA
		Normale Bewegung, keine Lahmheit erkennbar	0
		steifer Gang, geht langsam, Range of Motion eingeschränkt, keine eindeutige Lahmheit, (+/- Wendeschmerz)	2
		Lahmheit eindeutig sichtbar	4

This is a provisional file, not the final typeset article

Supplementary figure 3: Predicative Performance and discriminative power for longitudinal health outcomes of the refined MPS without the item lameness

A) The MPS without the lameness item showed good discriminative power for longitudinal health outcomes as demonstrated by the significant difference (p=0.0331) in the MPS scores of horses needing veterinary interventions (Y, red) compared to those that did not (N, black).

B) The ROC curve plots 100%-Specificity% vs. Sensitivity% for each MPS value. Using a cut-off for the MPS >4 yields a sensitivity of 67.74% and a specificity of 58.62% with a Youden index of 0.264 (p=0.034) for identifying horses in need of veterinary intervention (analgesia or euthanasia for unrelenting pain). Thus, horses with an MPS score above 4 without the lameness item have a high likelihood of a painful condition and should be further examined.

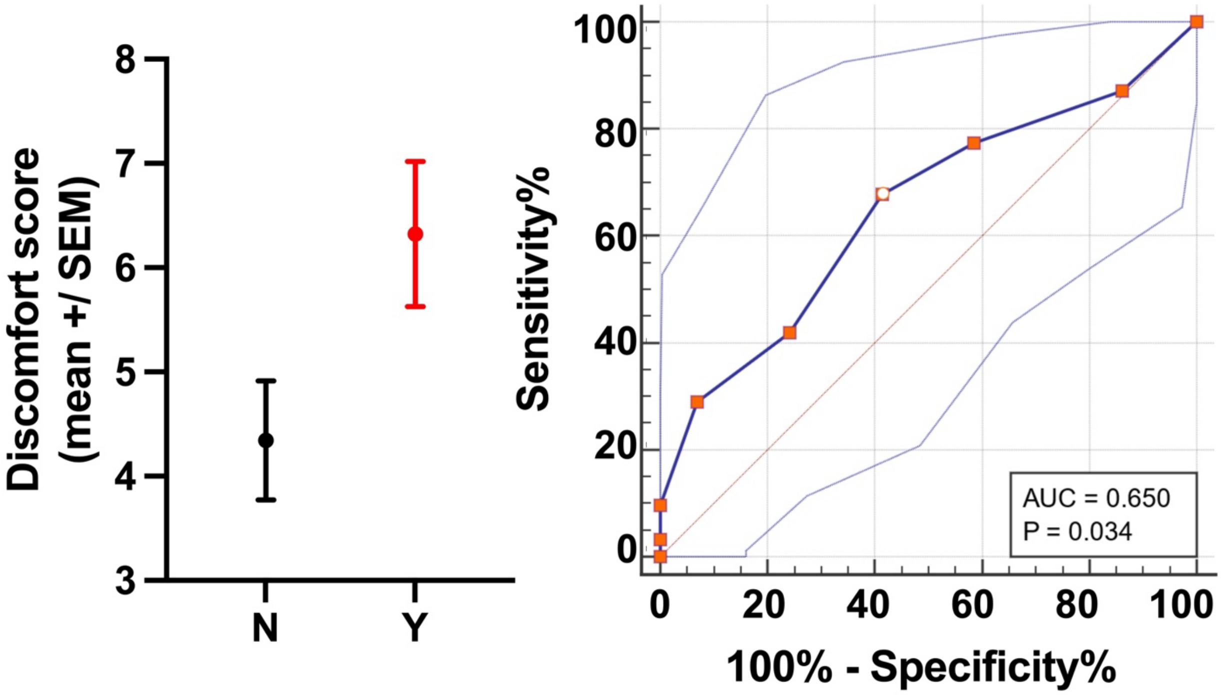

Supplementary table 1: Interrater agreement calculated by intraclass correlation analysis. During the refinement process, the item posture/weight distribution of the original score was divided into the items limb posture, weight shifting and weight bearing in the refined MPS.

	Original MPS		Refined MPS	
	ICC score	Conf. Interval	ICC score	Conf. Interval
Overall score	0.7743	0.62 - 0.88	0.88	0.80 – 0-94
Demeanour	0.14	-0.47 - 0.55	0.49	0.13 – 0.73
Pain face	0.66	0.41 - 0.83	0.56	0.26 – 0.77
Head-neck posture	0.73	0.55 – 0.86	0.74	0.55 – 0.86
Posture/weight distribution	0.69	0.46 – 0.84		
Limb posture			0.68	0.45 – 0.83
Weight shifting			0.88	0.79 – 0.94
Weight bearing			0.56	0.23 – 0.77
Lameness at the walk	0.83	0.71 – 0.91	0.86	0.71 – 0.93

This is a provisional file, not the final typeset article

Supplementary table 2: Sensitivity and specificity plus 95% confidence interval (CI) calculated by ROC analysis for each cut-off value for identifying horses needing veterinary intervention for the 7 MPS items: demeanour (c-statistic: 0.52, standard error: 0.075, p=0.82), pain face (c-statistic: 0.64, standard error: 0.071, p=0.06), head-neck posture (c-statistic: 0.5, standard error: 0.075, p=0.97), limb posture (c-statistic: 0.57, standard error: 0.075, p=0.39), weight shifting (c-statistic: 0.55, standard error: 0.75, p=0.52), weight bearing (c-statistic: 0.58, standard error:0.074, p=0.30) and lameness (c-statistic: 0.78, standard error: 0.06, p=0.0003).

Cut-off	Sensitivity (%)	95% CI	Specificity (%)	95% CI	Likelihood ratio
**MPS item demeanour**					
< 1.0	100	89.0% to 100%	3.45	0.18% to 17.2%	1.04
**MPS item pain face**					
> 1.0	58.1	40.8% to 73.6%	65.5	47.3% to 80.1%	1.68
> 3.0	19.4	9.19% to 36.3%	96.6	82.8% to 99.8%	5.61
**MPS item head-neck posture**					
> 1.0	41.9	26.4% to 59.2%	58.6	40.7% to 74.5%	1.01
**MPS item limb posture**					
> 1.0	87.1	71.1% to 94.9%	27.6	14.7% to 45.7%	1.2
> 3.0	48.4	32.0% to 65.2%	58.6	40.7% to 74.5%	1.17
> 5.0	6.45	1.15% to 20.7%	93.1	78.0% to 98.8%	0.94
**MPS item weight shifting**					
> 2.0	9.68	3.35% to 24.9%	100	88.3% to 100%	
**MPS item weight bearing**					
> 1.0	25.8%	13.7% to 43.2%	89.7	73.6% to 96.4%	2.49
> 3.0	9.68%	3.35% to 24.9%	96.6	82.8% to 99.8%	2.81
**MPS item lameness**					
> 1.0	77.4	60.2% to 88.6%	67.9	49.3% to 82.1%	2.41
> 3.0	38.7	23.7% to 56.2%	96.4	82.3% to 99.8%	10.8
